# ERK1 indicates good prognosis and inhibits breast cancer progression by suppressing YAP1 signaling

**DOI:** 10.18632/aging.102572

**Published:** 2019-12-18

**Authors:** Shiyi Yu, Meng Zhang, Ling Huang, Zhifang Ma, Xue Gong, Weiguang Liu, Jun Zhang, Liming Chen, Zhenghong Yu, Weiyong Zhao, Yan Liu

**Affiliations:** 1 Jiangsu Key Laboratory for Molecular and Medical Biotechnology, College of Life Science, Nanjing Normal University, Nanjing 210023, P.R. China; 2 Women’s Hospital of Nanjing Medical University, Nanjing Maternity and Child Health Care Hospital, Nanjing 210004, P.R. China; 3 Department of Medical Oncology, Jinling Hospital, Medical School of Nanjing University, Nanjing 210002, P.R. China; 4 Department of Oncology, Tongren Hospital, Shanghai Jiao Tong University, School of Medicine, Shanghai 200336, P.R. China; 5 The Key Laboratory of Bio-Medical Diagnostics, Suzhou Institute of Biomedical Engineering and Technology, Chinese Academy of Sciences, Suzhou 215163, P.R. China

**Keywords:** ERK1, ERK2, YAP1, breast cancer

## Abstract

The mitogen-activated protein kinase/extracellular signal-regulated (MAPK/ERK) pathway is a well-characterized signaling pathway during the development of various cancer types. ERK1 and ERK2, the two kinase effectors of MAPK cascade, exhibit high similarity. However, it is still unknown whether these two kinases are functionally different or in contrast functionally redundant during the development of breast cancer. We found that ERK1 expression levels were significantly lower in basal breast cancer compared with luminal breast cancer and normal breast tissues. RNA sequencing data suggested that ERK1 was associated with Hippo signaling pathway and cell proliferation in breast cancer cells. The gene set enrichment analysis (GSEA) further showed enrichment for YAP1 signaling pathway in breast cancer cell lines and tumors with low expression of ERK1. Silencing of ERK1 elevated YAP1 expression and TEAD activity in breast cancer cells. Additionally, ERK1 inhibited breast cancer cell proliferation via regulation of YAP1. The Kaplan-Meier analysis of data in patients with breast cancer suggested that, higher expression of ERK1 was associated with better prognosis, whereas, higher expression of ERK2 predicted poorer prognosis. These findings unveiled the role of ERK1 on regulation of YAP1 signaling pathway, indicating ERK1 as a negative regulator of breast cancer progression.

## INTRODUCTION

Breast cancer was the leading cause of women death with more than 2,000,000 new cases and 600,000 deaths in 2018 [1]. In recent decades, targeted therapies and molecules targeting specific signal transducers in pathways to interfere with a variety of oncogenic cellular processes, have been regarded as the future trend of cancer treatment [2].

The four mitogen-activated protein kinase (MAPK) cascades have been widely studied, which are named according to MAPK tier component: the extracellular signal-related kinases (ERK1/2), Jun amino-terminal kinases (JNK1/2/3), p-38 MAPK and ERK5 in mammalian cell [3]. These cascades consist of three protein kinases: a MAPK kinase kinase (MAP3K), a MAPK kinase (MAP2K) and a MAPK [4]. Numerous studies have paid close attention to the Ras/Raf/MEK/ERK1/2 signaling pathway due to its well established central role in mediating cancer cell proliferation [5–7]. Upon activation, ERK1/2 is translocated to the nucleus to phosphorylate and regulate various transcription factors, such as Ets family transcription factors (e.g., Elk-1), ultimately controlling gene expression [8]. ERK1 and ERK2 share 84% sequence in human [9]. Consistent with their high similarity in structure, experimental studies contend that ERK1 and ERK2 are functionally redundant, as observed during mouse development [10]. However, several studies have showed distinct functions between ERK1 and ERK2. For example, in NIH3T3, ERK2 mediated Ras-dependent cell proliferation, while ERK1 inhibited cell proliferation in a kinase activity independent manner [11].

The Hippo signaling pathway, an evolutionarily conserved pathway, plays a crucial role in cell proliferation, apoptosis, differentiation, as well as cancer development [12]. Yes-associated protein1 (YAP1), a transcriptional co-activator, is a downstream target of the Hippo signaling pathway [13]. When the Hippo signaling pathway is on, inactive YAP that is phosphorylated by upstream regulators is retained in the cytoplasm via binding to 14-3-3 and is degraded in a ubiquitin-proteasome-dependent manner [14, 15]. In contrast, active YAP1 is translocated from cytoplasm to nucleus and interacts with TEAD family to induce target expression [16]. In cancers, the abnormal expression of YAP1 causes uncontrolled cell growth and is connected with development of several cancer types including breast cancer [17–19].

Recent studies have reported that there is a crosstalk between Hippo-YAP pathway and MAPK-ERK pathway. Later, Wen Y et al found that the gene expression which took part in the phosphorylation of ERK1/2, was inhibited when YAP was silenced, and the protein levels of ERK and its downstream proteins were also reduced [20].

As for current study, we are the first to report the differences of distinct expression pattern, biological function and prognostic value between ERK1 and ERK2 in breast cancer. The *in vitro* and *in vivo* assays suggested that ERK1 inhibited breast cancer progression via downregulation of YAP1. Importantly, higher expression of ERK1 was correlated with better prognosis in patients with breast cancer and a predictor of better overall survival in patients receiving endocrine therapy. Higher expression of ERK2, in contrary, was associated with poorer prognosis.

## RESULTS

### Analysis of ERK1 and ERK2 expression in breast cancer cell lines and tumors

**Figure 1:**
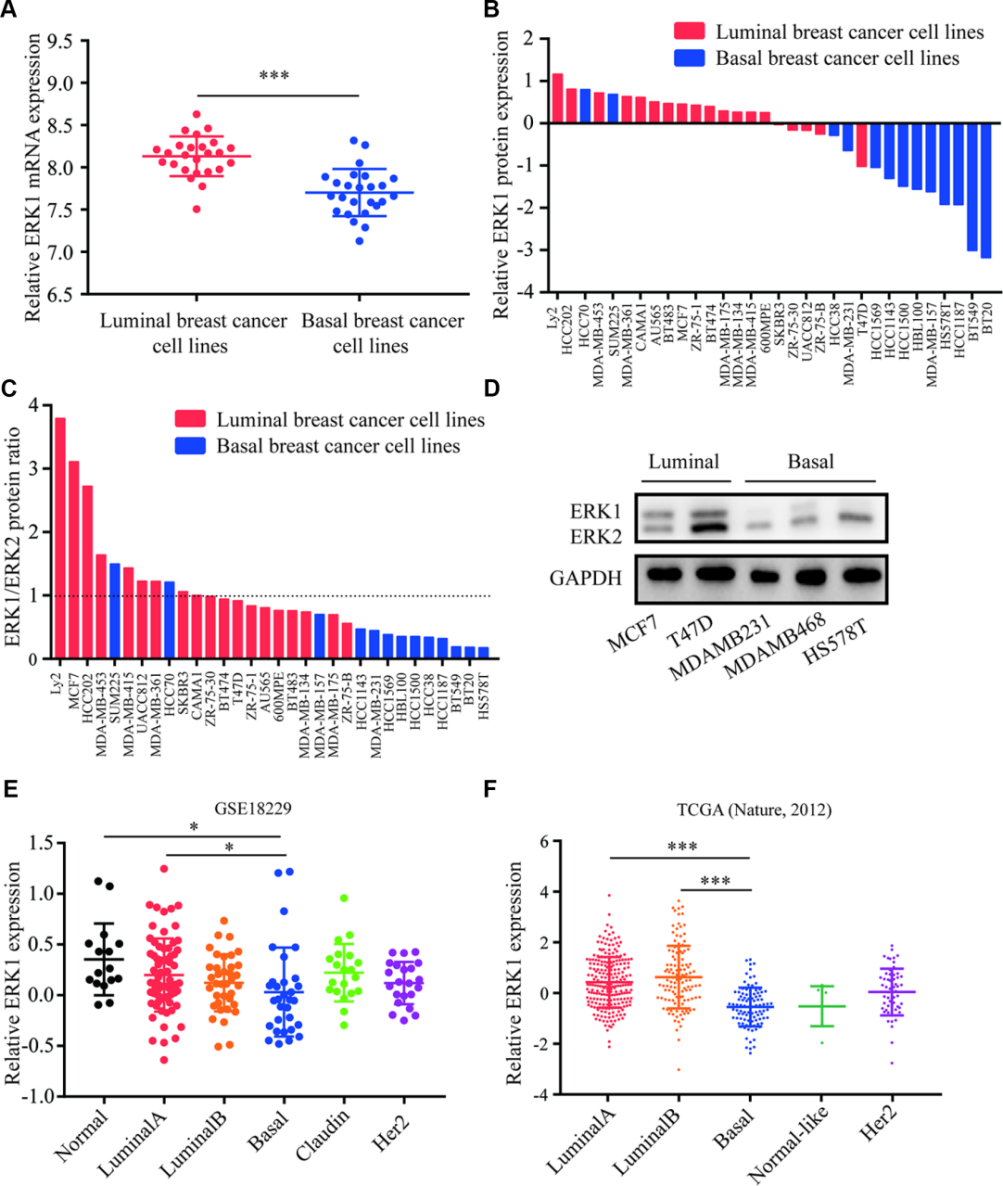
**Expression of ERK1 and ERK2 in breast cancer.** (**A**) Analysis of data for 50 breast cancer cell lines suggested that ERK1 mRNA was elevated in luminal breast cancer subtype when compared with basal breast cancer subtype. (**B**) Analysis of western blotting data from 32 breast cancer cell lines suggested that ERK1 protein expression was lower in basal breast cancer subtype when compared with luminal breast cancer subtype. (**C**) Analysis of western blotting data from 32 breast cancer cell lines suggested that ERK1/ERK2 protein expression ratio was lower in basal breast cancer subtype when compared with luminal breast cancer subtype. (**D**) Western blotting confirmed that ERK1 was highly expressed in luminal breast cancer cell lines compared with basal breast cancer cell lines tested. (**E**) Analysis of GSE18229 dataset for 16 normal breast tissues and 180 breast tumors suggested that ERK1 was downregulated in basal breast cancer tumors when compared with Luminal A breast cancer subtype and normal breast tissues. (**F**) Analysis of TCGA dataset for 519 breast tumors showed that ERK1 was downregulated in basal breast cancer tumors when compared with Luminal A breast cancer subtype and Luminal B breast cancer subtype. *, p<0.05; ***, p<0.001.

Expression data for 50 breast cancer cell lines were downloaded from a previous study [38]. The cell lines were then divided into luminal breast cancer subtype and basal breast cancer subtype, in which the expression of ERK1 and ERK2 were analyzed. Higher expression of ERK1 was observed in luminal breast cancer cell lines when compared with basal breast cancer cell lines (Figure 1A), whereas the ERK2 expression was not associated with breast cancer subtype (Supplementary Figure 1A). For analysis of ERK1 and ERK2 protein expression pattern, the western blot data of 32 breast cancer cell lines (19 luminal breast cancer cell lines and 13 basal breast cancer cell lines) [38] were also analyzed. It was observed that ERK1 protein was highly expressed in luminal breast cancer cell lines compared with basal breast cancer (Figure 1B), ERK2 protein levels were similar between the two subtypes (Supplementary Figure 1B). In addition, the ratio of ERK1 to ERK2 protein expression was also higher in luminal breast cancer cell lines than in basal breast cancer cell lines (Figure 1C). Using western blotting, we confirmed relative higher expression of ERK1 in luminal breast cancer cell lines (MCF7 and T47D) as compared to basal breast cancer cell lines (MDA-MB-231, BT549 and HS578T) (Figure 1D). Data for 16 normal breast samples and 180 tumor samples were downloaded from GEO (GSE18229). It was observed that ERK1 was decreased in basal breast cancer subtype when compared with normal breast cancer subtype and luminal breast cancer subtype (Figure 1E). ERK2 expression was similar among different breast cancer subtype (Supplementary Figure 1C). This observation was further evaluated in a large cohort. Via analysis of ERK1 expression in TCGA dataset (519 cases), basal breast cancer tumors exhibited lower expression of ERK1 in comparison with luminal A and luminal B breast cancer subtypes (Figure 1F). Interestingly, ERK2 expression levels were elevated in basal breast cancer subtype compared with luminal breast cancer subtype (Supplementary Figure 1D).

### Changes in gene expression in breast cancer cells upon knockdown of ERK1

**Figure 2:**
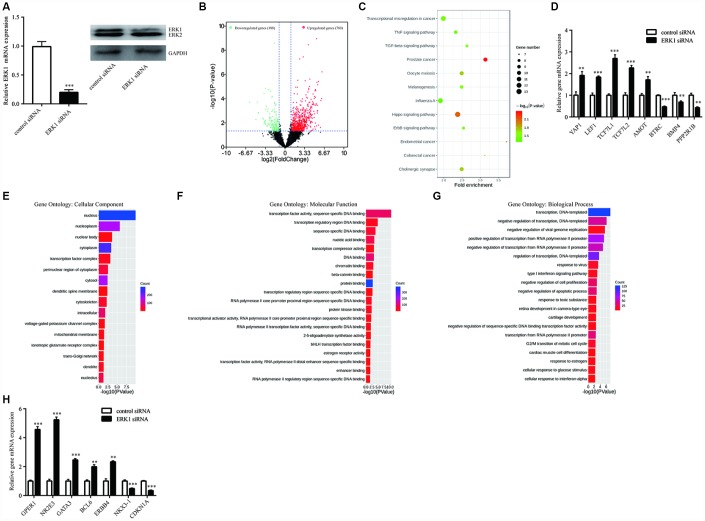
**RNA sequencing (RNAseq) analysis of ERK1-silenced T47D cells.** (**A**) RT-qPCR and western blotting showed that ERK1 siRNA decreased ERK1 mRNA and protein expression in T47D cells. (**B**) Volcano plot showed that ERK1 silencing increased a set of 780 genes in abundance of log2FC ≥ 1, while a set of 188 genes decreased in abundance of log2FC ≤ -1, based on transcriptome sequencing of control group and ERK1 siRNA group. (**C**) KEGG pathway enrichment analysis of differentially expressed genes in ERK1 siRNA group compared to control group. (**D**) Validation of identified genes in “Hippo signaling pathway”, including YAP1, LEF1, TCF7L1, TCF7L2, AMOT, BTRC, BMP4 and PPP2R1B. (**E**) Gene Ontology annotation analysis of the significantly enriched cellular component (P < 0.05). (**F**) Gene Ontology annotation analysis of the top 20 significantly enriched Molecular Function (P < 0.05) that changes in gene expression. (**G**) Gene Ontology annotation analysis of the top 20 significantly enriched Biological Process (P < 0.05) that changes in gene expression. H. Validation of genes in “negative regulation of cell proliferation”, including GPER1, NR2E3, GATA3, BCL6, ERBB4, NKX3-1 and CDKN1A. **, p<0.01; ***, p<0.001.

To study the global gene transcription regulated by ERK1, RNA sequencing (RNA-Seq) analysis was utilized to determine global gene expression change upon knockdown of ERK1 in T47D cells (Figure 2A). ERK1 knockdown significantly modulated 968 (188 downregulated and 780 upregulated) genes (Figure 2B). Supplementary Table 1 listed the differentially expressed genes in T47D cells. The KEGG enrichment analysis indicated that ERK1 was involved in several cancer-related signaling pathways, such as Hippo signaling pathway, ErbB signaling pathway and TNF signaling pathway (Figure 2C). Several key genes in Hippo signaling pathway were modulated after ERK1 knockdown including YAP1, transcription factors that interacted with YAP1 (LEF1, TCF7L1, TCF7L2) and target genes of YAP1 (BBC3). We validated the differentially expressed genes (YAP1, LEF1, TCF7L1, TCF7L2, AMOT, BTRC, BMP4, PPP2R1B) at the mRNA levels (Figure 2D). Further GO analysis of Cellular Component showed that nucleus genes were significantly enriched upon ERK1 knockdown (Figure 2E). GO analysis of Molecular Functions showed that several DNA binding and transcription related genes were enriched upon ERK1 knockdown, the top 20 enriched gene sets were listed in Figure 2F. We also performed GO analysis of Biological Process. Among the top 20 enriched processes, genes of cell proliferation (negative regulation of cell proliferation), cycle (G2/M transition of mitotic cell cycle) and apoptosis (negative regulation of apoptotic process) were enriched (Figure 2G). Several breast cancer related oncogenes (GPER1, NR2E3, GATA3, BCL6, ERBB4) were upregulated, while tumor suppressors (NKX3-1, CDKN1A) were downregulated, which were validated with RT-qPCR (Figure 2H).

### ERK1 expression was associated with YAP1 signaling-related gene expression in breast cancer cell lines

**Figure 3:**
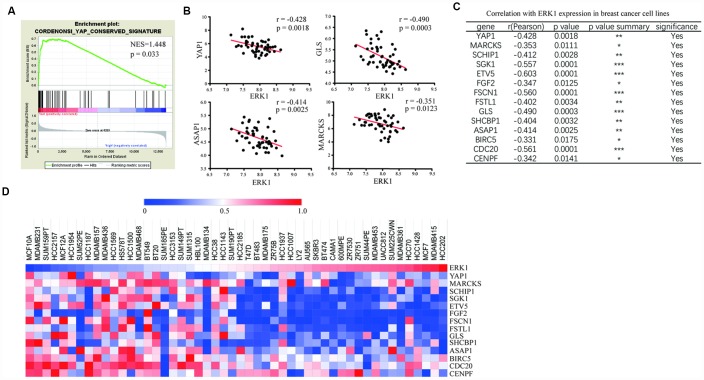
**ERK1 was negatively associated with YAP1 signaling genes in breast cancer cells.** (**A**) GSEA of expression data from breast cancer cell lines revealed enrichment of conserved YAP1 target genes in ERK1 low expression cell lines compared with those with high ERK1 expression. NES, normalized enrichment score. (**B**) Pearson correlation analysis showed that ERK1 expression levels were negatively correlated with YAP1 and its target gene expression (GLS, ASAP1, MARCKS) in 50 breast cancer cell lines analyzed. (**C**) List of the Pearson analysis of correlation between several YAP1 target genes and ERK1 in 50 breast cancer cell lines. (**D**) Heat map showing low expression levels of ERK1 enriched high expression of YAP1 signaling-related genes in breast cancer cell lines.

To investigate the association between ERK1 and YAP1 signaling, we analyzed expression of evolutionarily conserved signature of the YAP1 signaling genes described by Cordenonsi et al [21] with ERK1. Using transcriptome data of 50 breast cancer cell lines, the GSEA revealed strong enrichment of YAP1 signaling-related genes in ERK1 low expression breast cancer cell lines compared with those with high ERK1 expression (Figure 3A). We further extended this finding by analysis the correlation between expression of ERK1 and YAP1 signaling-related genes, which revealed a strong negative correlation between them (Figure 3B, 3C). The heatmap analysis suggested that low expression of ERK1 enriched high expression of YAP1 and its target genes in breast cancer cells (Figure 3D).

### ERK1 negatively regulated the activation of YAP1 signaling pathway

**Figure 4:**
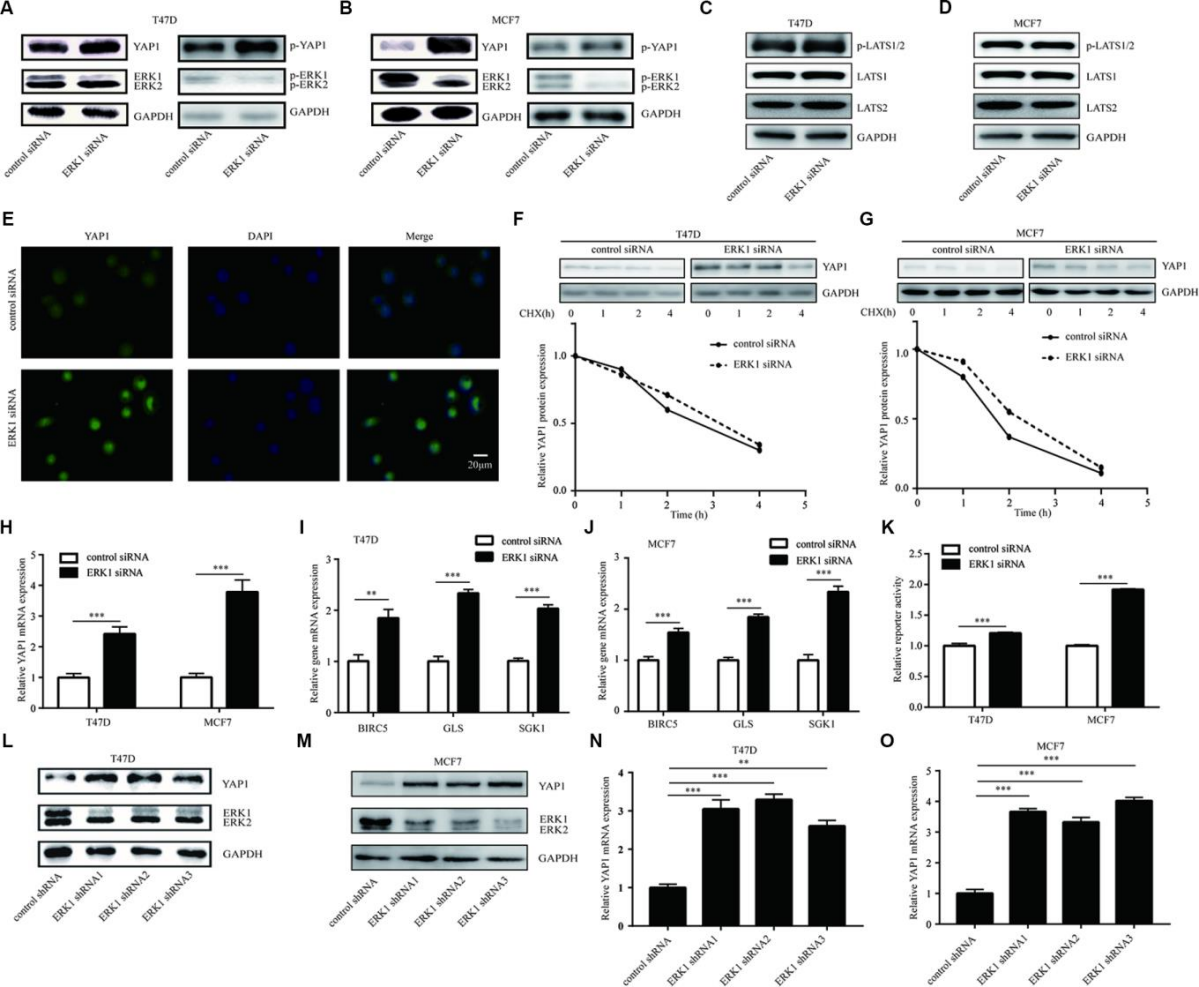
**ERK1 repressed YAP1 signaling-related gene expression in breast cancer cells.** (**A**) Western blotting showed that silencing of ERK1 increased YAP1 protein levels in T47D cells. (**B**) Western blotting showed that silencing of ERK1 increased YAP1 protein levels in MCF7 cells. (**C**) Western blotting showed that silencing of ERK1 did not change p-LATS1/2, LATS1 and LATS2 protein levels in T47D cells. (**D**) Western blotting showed that silencing of ERK1 did not change p-LATS1/2, LATS1 and LATS2 protein levels in MCF7 cells. (**E**) Immunofluorescence showed that ERK1 silencing increased YAP1 protein expression in T47D cells. (**F**) The CHX chase assay showed that the YAP1 protein stability was not altered upon silencing of ERK1 in T47D cells. (**G**) The CHX chase assay showed that the YAP1 protein stability was not altered upon silencing of ERK1 in MCF7 cells. (**H**) RT-qPCR showed that ERK1 silencing elevated YAP1 mRNA levels in T47D cells and MCF7 cells. (**I**) RT-qPCR showed that ERK1 silencing elevated mRNA levels of YAP1 downstream genes (BIRC5, GLS, SGK1) in T47D cells. (**J**) RT-qPCR showed that ERK1 silencing elevated mRNA levels of YAP1 downstream genes (BIRC5, GLS, SGK1) in MCF7 cells. (**K**) Knockdown of ERK1 increased GTIIC reporter activity in T47D cells and MCF7 cells. (**L**) Lentivirus mediated knockdown of ERK1 increased YAP1 protein expression in T47D cells. (**M**) Lentivirus mediated knockdown of ERK1 increased YAP1 protein expression in MCF7 cells. (**N**) Lentivirus mediated knockdown of ERK1 increased YAP1 mRNA expression in T47D cells. (**O**) Lentivirus mediated knockdown of ERK1 increased YAP1 mRNA expression in MCF7 cells. *, p<0.05; **, p<0.01; ***, p<0.001.

To validate the aforementioned observation, we silenced ERK1 in breast cancer cells. Western blotting showed that ERK1 knockdown increased the protein expression of YAP1 and phosphorylated YAP1 in luminal breast cancer cells, especially in MCF7 cells with higher ERK1 protein level (Figure 4A, 4B). ERK1 knockdown did not changed the expression of LATS1, LATS2 and p-LATS1/2 in T47D and MCF7 cells (Figure 4C, 4D), suggested that the kinase cassette of Hippo pathway was not affected. Immunofluorescence showed that ERK1 silencing increased YAP1 expression without inducing a translocation of YAP1 protein in T47D cells (Figure 4E), suggesting that the activation of Hippo pathway was not involved in this process. The CHX chase assay further confirmed that the stability of YAP1 was not changed in T47D cells or MCF7 cells after ERK1 silencing (Figure 4F, 4G). RT-qPCR suggested that knockdown of ERK1 by ERK1 siRNA elevated YAP1 mRNA levels in T47D cells and MCF7 cells (Figure 4H). Furthermore, RT-qPCR showed that ERK1 silencing elevated the expression of YAP1 downstream genes (BIRC5, GLS, SGK1) in T47D and MCF7 cells (Figure 4I, 4J). After ERK1 knockdown, the activity of GTIIC plasmid decreased in T47D cells and MCF7 cells (Figure 4K), indicating the decreased activity of TEAD-YAP1 complex. We next used lentivirus mediated knockdown of ERK1 to validate the regulatory association between ERK1 and YAP1. Knockdown of ERK1 by three target-specific shRNAs increased YAP1 protein levels in T47D cells (Figure 4L). The similar results were also observed in MCF7 cells (Figure 4M). RT-qPCR further showed ERK1 shRNAs increased YAP1 mRNA expression in T47D and MCF7 cells (Figure 4N, 4O). Our previous study indicated that silencing of both ERK1 and ERK2 decreased YAP1 expression in cancer cells [37]. To examine how ERK2 regulated YAP1, ERK2 was silenced in T47D cells. Interestingly, in contrast to ERK1 silencing, ERK2 silencing decreased YAP1 protein expression (Supplementary Figure 2A). The downregulation of YAP1 by ERK2 silencing was further observed in basal breast cancer cells (MDA-MB-231 and HS578T) (Supplementary Figure 2B). The CHX chase assay showed that ERK2 silencing destabilized YAP1 protein (Supplementary Figure 2C). The YAP1 mRNA expression was not changed in T47D cells or MDA-MB-231 cells after ERK2 silencing (Supplementary Figure 2D).

### ERK1 inhibited breast cancer cell proliferation by downregulation of YAP1

**Figure 5:**
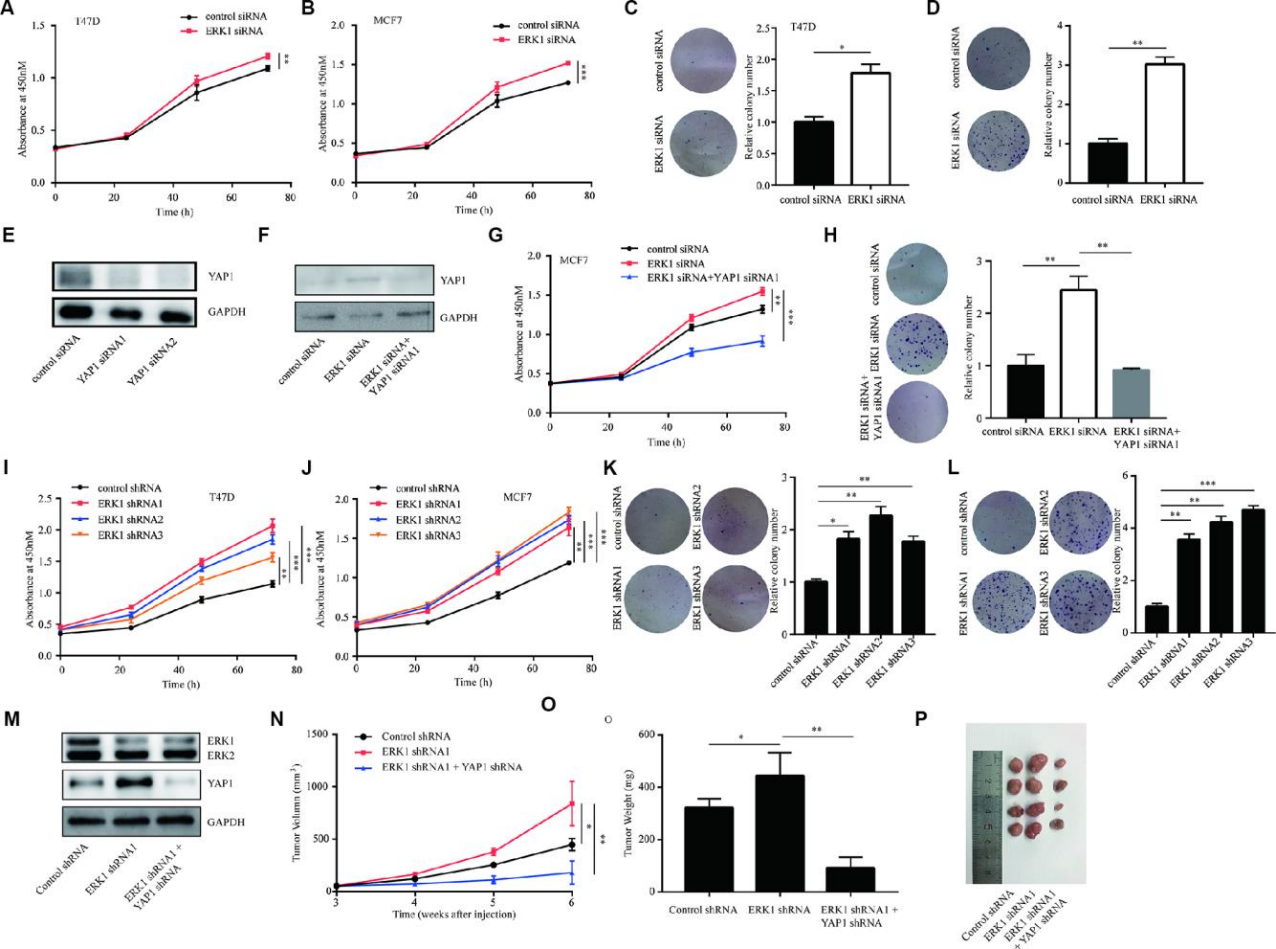
**ERK1 promoted cell proliferation of breast cancer cells *in vitro* and *in vivo.*** (**A**) In T47D cells, silencing of ERK1 increased cell proliferation ability. (**B**) In MCF7 cells, silencing of ERK1 increased cell proliferation ability. (**C**) In T47D cells, silencing of ERK1 increased colony forming ability. (**D**) In MCF7 cells, silencing of ERK1 increased colony forming ability. (**E**) Transfection of YAP1 siRNA decreased YAP1 protein expression in MCF7 cells. (**F**) Silencing of YAP1 reversed ERK1 silencing induced elevation of YAP1 protein expression in MCF7 cells. (**G**) Silencing of YAP1 reversed ERK1 silencing induced elevation of cell proliferation ability in MCF7 cells. (**H**) Silencing of YAP1 reversed ERK1 silencing induced elevation of colony forming ability in MCF7 cells. (**I**) The cell proliferation of T47D cells with stable knockdown of ERK1 was increased in comparison with T47D cells infected with control shRNA. (**J**) The cell proliferation of MCF7 cells with stable knockdown of ERK1 was increased in comparison with MCF7 cells infected with control shRNA. (**K**) The colony forming ability of T47D cells was decreased after lentivirus mediated ERK1 knockdown. (**L**) The colony forming ability of MCF7 cells was decreased after lentivirus mediated ERK1 knockdown. (**M**) Western blotting showed that lentivirus mediated knockdown of ERK1 decreased ERK1 protein expression and elevated YAP1 protein expression, while knockdown of both ERK1 and YAP1 decreased ERK1 and YAP1 protein expression in MCF7 cells. (**N**) Xenografted tumor growth curve indicated that ERK1 knockdown increased tumor volume, while YAP1 knockdown decreased tumor volume *in vivo*. (**O**) ERK1 knockdown increased tumor weight, while YAP1 knockdown decreased tumor weight *in vivo*. (**P**) Representative xenografted tumors from nude mouse models. *, p<0.05; **, p<0.01; ***, p<0.001.

In above, we have reported the effects of ERK1 in regulation of YAP1. To further characterize the function of ERK1, we detected cell proliferation ability after silencing ERK1 in breast cancer cell lines. In T47D and MCF7 cells, silencing of ERK1 enhanced cell proliferation ability (Figure 5A, 5B). Additionally, ERK1 silencing increased colony forming ability in T47D and MCF7 cells (Figure 5C, 5D). To determine whether ERK1 regulated cell proliferation via controlling YAP1 expression, we silenced YAP1 protein expression in MCF7 cells (Figure 5E). In the rescue assays, silencing of YAP1 reversed ERK1 siRNA mediated upregulation of YAP1 as well as the increase of cell proliferation and colony formation ability in MCF7 cells (Figure 5F, 5H). We further found that lentivirus mediated knockdown of ERK1 promoted cell proliferation in T47D and MCF7 cells (Figure 5I, 5J). The colony forming ability of T47D and MCF7 cells were also elevated following lentivirus mediated knockdown of ERK1 (Figure 5K, 5L). Consistent with the *in vitro* experiments, in xenograft mouse models, silencing of ERK1 increased tumor volume and weight after injection of MCF7 cells, which was reversed by silencing of YAP1 (Figure 5M–5P). The results demonstrated that ERK1 inhibited the growth of breast cancer cells and tumors via downregulation of YAP1.

### ERK1 was associated with expression of YAP1 signaling-related genes in breast cancer tumors

**Figure 6:**
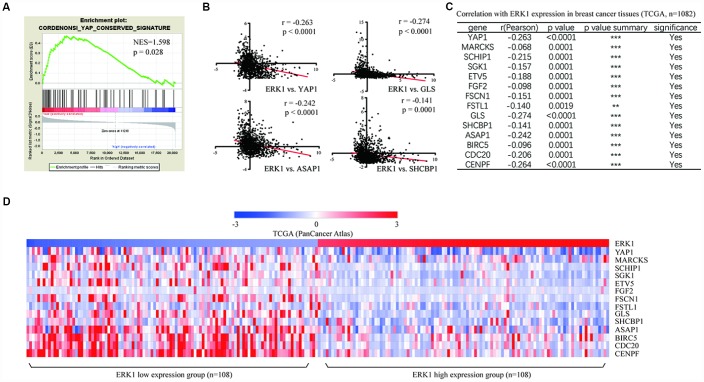
**ERK1 was negatively associated with YAP1 signaling-related gene in breast tumors.** (**A**) GSEA of expression data from breast cancer cell lines revealed enrichment of conserved YAP1 target genes in ERK1 low expression tumors compared with those with high ERK1 expression by quartiles. NES, normalized enrichment score. (**B**) Pearson correlation analysis showed that ERK1 expression levels were negatively correlated with YAP1 and its target gene expression (GLS, ASAP1, SHCBP1) in 1,082 breast tumors. (**C**) List of the Pearson analysis of correlation between several YAP1 target genes and ERK1 in 1082 breast tumors. (**D**) Heat map showing low expression levels of ERK1 (top 10% ERK1 high expression cases vs. 10% ERK1 low expression cases) enriched YAP1 signaling-related gene expression in TCGA dataset containing 1082 cases.

To further evaluate the association between ERK1 and YAP1 signaling pathway in clinical samples, expression data of 1082 breast cancer tumors were downloaded from TCGA (PanCancer Atlas). Samples were categorized by expression level that was above (high) or below (low) median ERK1 expression by quartiles (271 samples each group). The GSEA revealed a strong enrichment of YAP1 signaling-related genes in ERK1 low expression tumors compared with those with high ERK1 expression (Figure 6A). Furthermore, correlation analysis showed negative correlation between ERK1 and YAP1 signaling-related genes (Figure 6B). Among multiple YAP1 signaling-related genes, a majority of them (32 out of 58) were negatively correlated with ERK1 expression, as some of them were showed in Figure 6C. It was observed that, there was lower expression of YAP1 signaling-related genes in ERK1 high expression group in comparison with ERK1 low expression group (Figure 6D).

### Expression of ERK1 and ERK2 were associated with prognosis of patients with breast cancer

**Figure 7:**
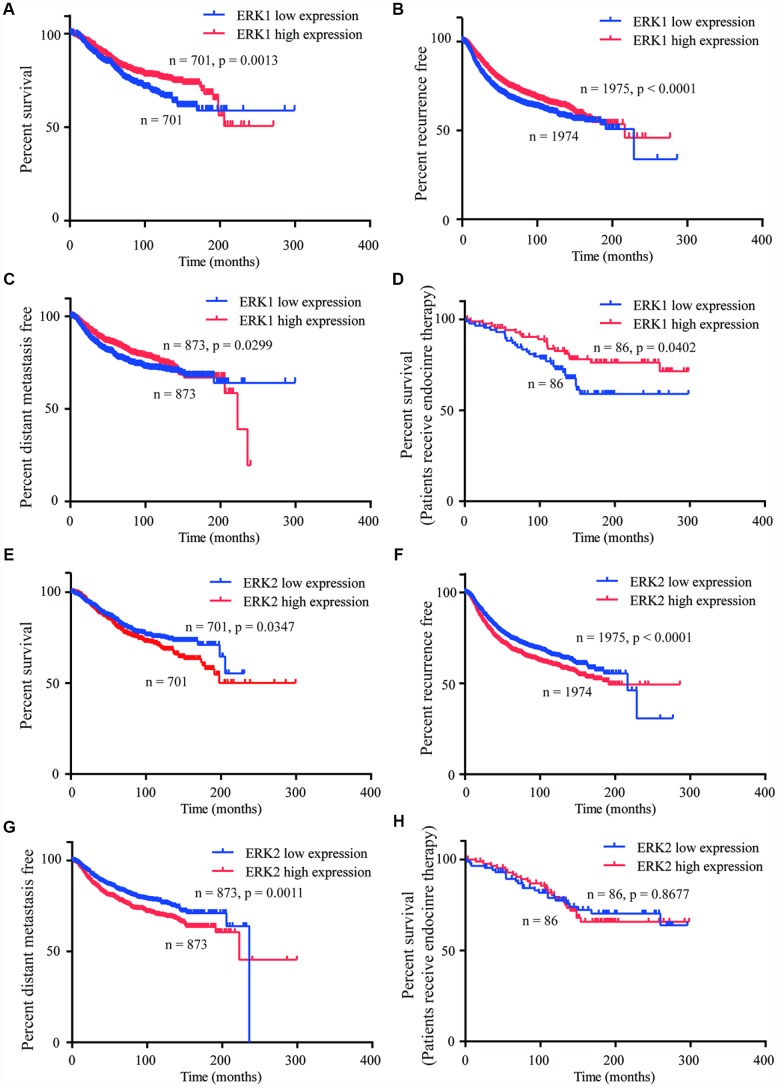
**ERK1 and ERK2 expression were associated with distinct prognostic features of breast cancer.** (**A**–**C**) Kaplan-Meier Plotter analysis indicated that high expression of ERK1 was associated with prolonged OS, recurrence and DMFS in patients with breast cancer. (**D**) Kaplan-Meier Plotter analysis indicated that high expression of ERK1 was associated with prolonged OS in patients receiving endocrine therapy. (**E**–**G**) Kaplan-Meier Plotter analysis showed that the high expression of ERK2 was associated with poor OS, recurrence and DMFS in patients with breast cancer. (**H**) Kaplan-Meier Plotter analysis indicated that high expression of ERK2 was not associated with OS in patients receiving endocrine therapy.

YAP1 signaling was pivotal for cancer cell proliferation, metastasis and drug resistance [12]. The Kaplan-Meier Plotter was used to analyze the association between ERK1/ERK2 and the prognosis of patients with breast cancer. Consistently, high expression of ERK1 was associated with prolonged overall survival (OS) (Figure 7A), recurrence free (Figure 7B) and distant metastasis free survival (DMFS) (Figure 7C) in patients with breast cancer. High expression of ERK1 also predicted good OS in patients receiving endocrine therapy (Figure 7D). In contrast, high expression of ERK2 was associated with poor OS (Figure 7E), recurrence free (Figure 7F) and DMFS (Figure 7G) in patients with breast cancer. However, the ERK2 expression was not associated with OS in patients receiving endocrine therapy (Figure 7H).

## DISCUSSION

In 2006, Vantaggiato et al. discovered that transfection of ERK1 instead of ERK2 could inhibit transformation of NIH3T3 induced by stable expression of oncogenic Ras [11]. Later, several studies provided evidences supporting the hypothesis of ERK1 and ERK2 exhibiting functional redundancy [22]. In this article, the specific roles of ERK1 and ERK2 in regulation of YAP1 signaling in breast cancer were studied.

Previously, we discovered a crosstalk between the MAPK/ERK pathway and YAP1 signaling in cancer cells [37]. Our findings were further validated in several following studies. For example, Lei Q et al found that the MAPK–ERK–YAP axis was involved in MA-5-mediated Bnip3 upregulation and mitophagy activation [23]. One study in glioblastoma showed that TNFα stimulation decreased p-ERK expression, led to reduction of YAP1 expression [24]. In primary hPDLF cells, inhibition of ERK decreased total YAP amount in biomechanical strained cells but did not influence distribution of YAP1 in nuclear or cytoplasm [25]. In lung cancer, silencing or inhibition of ERK1/2 led to downregulation of YAP1 protein and YAP1 downstream genes [26]. In current study, we focused on the specific roles of ERK1 and ERK2 on regulation of YAP1 in breast cancer. Consistent with ERK inhibition and ERK1/2 silencing, ERK2 knockdown decreased YAP1 expression in MDA-MB-435S, which was further validated in two breast cancer cell lines. Our CHX chase assays further demonstrated that ERK2 but not ERK1 could stabilize YAP1 expression in cancer cells. However, we did not observe direct interaction between ERK2 and YAP1 (data not shown), suggesting that ERK2 might indirectly regulate the decay of YAP1 in cancer cells, which will be further explored in our future study.

A previous report of mouse embryo discovered that the total activity of ERK1/2 was important for mouse development, which was in support of the notion that ERK1 and ERK2 showed functional redundancy [10]. In contrast, in zebrafish embryo, ERK1 knockdown exhibited strong phenotypes at later stages in embryogenesis while ERK2 knockdown inhibited the embryo from entering epiboly [27]. Through analyzing gene expression data of breast cancer cell lines and tumors, we observed that ERK1 was significantly downregulated in basal breast cancer subtype compared with luminal subtype. It was known that basal breast cancer subtype was much more invasive and less differentiated compared with luminal breast cancer type [38]. In particular, the most recent studies have indicated that YAP1 and its target genes were overexpressed in basal breast cancer subtype and pivotal for maintaining cancer cell stemness and drug resistance [28, 29]. Following these notions, our bioinformatic analysis between ERK1 and YAP1 signaling-related gene demonstrated that ERK1 levels were negatively associated with YAP1 signaling-related gene levels in breast tumors and cell lines. The GSEA indicated that YAP1 signaling-related genes were enriched in breast cancer cell lines and tumors which expressed low ERK1 levels. Our following experiments confirmed that ERK1 negatively regulated YAP1 at mRNA level in breast cancer cells. Previous reports identified several non-coding RNAs as the regulator of YAP1 mRNA expression in cancer cells [30, 31]. Transcription factors and epigenetic modification were also discovered as direct regulators of YAP1 transcription [32, 33]. Further investigation is needed to explore how ERK1 control YAP1 mRNA expression.

YAP1 was crucial for breast cancer cell proliferation, metastasis and drug resistance [34], the distinct functions of ERK1 and ERK2 on regulation of YAP1 suggested distinct roles of ERK1 and ERK2 on breast cancer progression. The previous microarray study discovered that in addition to commonly affected genes, nearly half of ERK1 affected genes were specifically regulated by ERK1 [27]. Our KM plotter analysis showed that ERK1 overexpression was associated with good prognosis in patients with breast cancer, indicating a *bona fide* tumor suppressor role of ERK1 at least in breast cancer. High expression of ERK2, in contrast, was associated with poor prognosis in patients with breast cancer. It is known that basal breast cancer cells are particularly sensitive to RAF-MEK-ERK inhibitors compared with other subtypes, and ERK1 high expression predicted resistance to a MEK inhibitor [35]. Our findings on the distinct roles and expression pattern of ERK1 and ERK2 in breast cancer could provide a new understanding for the different susceptibility.

## CONCLUSIONS

In conclusion, our study demonstrated that ERK1 and ERK2 showed distinct expression pattern, prognostic value and function in regulation of YAP1 signaling in breast cancer. Our results supported a tumor suppressor role of ERK1 in breast cancer via suppression of YAP1 signaling pathway. Additional studies are needed to determine the molecular mechanism on regulation of YAP1 mRNA levels by ERK1; however, our data firstly suggested that ERK1 expression might be used as biomarker for prediction the prognosis in patients with breast cancer.

## MATERIALS AND METHODS

### Cell culture

293T cells, breast cancer cell lines MCF7, T47D, MDA-MB-231, BT549 and HS578T were purchased from American Type Culture Collection (ATCC, Manassas, VA). MDA-MB-435S, MDA-MB-231 and BT549 were cultured in DMEM (Life Technologies, Carlsbad, CA), supplemented with 10% FBS (Hyclone, Logan, UT). 293T, MCF7 and T47D were cultured in RPMI-1640 medium (Corning Cellgro) supplemented with 10% FBS (Hyclone). All cells were maintained in a humid incubator with 5% CO_2_ at 37°C

### RNA interference

ERK1 siRNA pool (a pool of 3 target-specific siRNA) (sc-44205), ERK2 siRNA pool (a pool of 3 target-specific siRNA) (sc-44223) and control siRNA (sc-37007) were purchased from Santa Cruz Biotechnology (Santa Cruz, CA). YAP1 siRNA was bought from GenePharma (Suzhou, China). SiRNAs were transfected into MDA-MB-435S, MCF7, T47D, MDA-MB-231, HS578T and BT549 cells using Lipofectamine RNAi MAX (Invitrogen, Carlsbad, CA) reagent according to manufacturer’s protocol. After 72 h, the cells were collected and subjected to the following experiments.

### RNA sequencing

Transcriptome libraries and RNA sequencing analysis were performed, aligned and normalized according to the Illumina Genome Analyzer II (Illumina, San Diego, CA, USA) manufacturer’s instruction. After clustering, the libraries were sequenced on an Illumina HiseqXTen platform using (2×150 bp) paired-end module. After initial quality control, the clean reads were mapped to the reference sequence by using TopHat2 software (v2.1.1.). The alignment files generated by TopHat2 were input to the Cufflinks software (v2.2.1), which was a program for the comparative assembly of transcripts and the estimation of their abundance in a transcriptome sequencing experiment by using the measurement unit fragments per kilobase of transcript per million mapped reads (FPKM). After using Cuffmerge program to merge transcripts of each sample in different materials and stages into a single gtf file that was used to identify differentially expressed genes, Cuffdiff program was used to find differentially expressed genes (DEGs). The differentially expressed genes were identified with p value ≤0.05 and a fold-change of ≥2. Database for Annotation, Visualization and Integrated Discovery (DAVID) was used to analyzed the Gene Ontology (GO) and Kyoto Encyclopedia of Genes and Genomes (KEGG) pathway modulated by the knockdown of ERK1 or ERK2 [36].

### RNA extraction and RT-qPCR

Total RNA of MDA-MB-435S, MCF7, T47D, HS578T and BT549 cells were extracted using TRIzol reagent (Invitrogen). The concentration and quality of RNA were measured with NanoDrop2000 (Thermo Fisher Scientific, Waltham, MA). The RNA was reverse transcribed into first-strand cDNA with PrimeScript RT reagent kit (TaKaRa, Otsu, Shiga, Japan). RT-qPCR was performed with SYBR Premix Ex Taq (TaKaRa) on a CFX96 Real-Time PCR System (Bio-rad, Hercules, CA). The primer sequences were listed in Table 1.

**Table 1:** RT-qPCR primer sequences.

**Primers**	**sequences**
ERK1-forward	5′-TAGGCATCCGAGACATCCTC-3′
ERK1-reverse	5′-AGCTGCTGGCTCTTTAGCAG-3′
ERK2-forward	5′-TACACCAACCTCTCGTACATCG-3′
ERK2-reverse	5′-CATGTCTGAAGCGCAGTAAGATT-3′
YAP1-forward	5′-TAGCCCTGCGTAGCCAGTTA-3′
YAP1-reverse	5′-TCATGCTTAGTCCACTGTCTGT-3′
BBC3-forward	5′-GACCTCAACGCACAGTACGAG-3′
BBC3-reverse	5′-AGGAGTCCCATGATGAGATTGT-3′
LEF1-forward	5′-AGAACACCCCGATGACGGA-3′
LEF1-reverse	5′-GGCATCATTATGTACCCGGAAT-3′
TCF7L1-forward	5′-TCGTCCCTGGTCAACGAGT-3′
TCF7L1-reverse	5′-ACTTCGGCGAAATAGTCCCG-3′
TCF7L2-forward	5′-AGAAACGAATCAAAACAGCTCCT-3′
TCF7L2-reverse	5′-CGGGATTTGTCTCGGAAACTT-3′
AMOT-forward	5′-AGGCAAGAGTTGGAAGGATGC-3′
AMOT-reverse	5′-AGGATGACTTCACGAGGTTCT-3′
BTRC-forward	5′-CCAGACTCTGCTTAAACCAAGAA-3′
BTRC-reverse	5′-GGGCACAATCATACTGGAAGTG-3′
BMP4-forward	5′-ATGATTCCTGGTAACCGAATGC-3′
BMP4-reverse	5′-CCCCGTCTCAGGTATCAAACT-3′
CDKN1A-forward	5′-TGTCCGTCAGAACCCATGC-3′
CDKN1A-reverse	5′-AAAGTCGAAGTTCCATCGCTC-3′
BCL6-forward	5′-GGAGTCGAGACATCTTGACTGA-3′
BCL6-reverse	5′-ATGAGGACCGTTTTATGGGCT-3′
PPP2R1B-forward	5′-CTTGTGTCAGTATTGCCCAGT-3′
PPP2R1B-reverse	5′-TGCTGCTTGTCGAAGTGTAGG-3′
GPER1-forward	5′-CACCAGCAGTACGTGATCGG-3′
GPER1-reverse	5′-CATCTTCTCGCGGAAGCTGAT-3′
NR2E3-forward	5′-AGCAGCGGGAAGCACTATG-3′
NR2E3-reverse	5′-CCTGGCACCTGTAGATGAGC-3′
ERBB4-forward	5′-GTCCAGCCCAGCGATTCTC-3′
ERBB4-reverse	5′-AGAGCCACTAACACGTAGCCT-3′
NKX3-1-forward	5′-CCCACACTCAGGTGATCGAG-3′
NKX3-1-reverse	5′-GAGCTGCTTTCGCTTAGTCTT-3′
GATA3-forward	5′-GCCCCTCATTAAGCCCAAG-3′
GATA3-reverse	5′-TTGTGGTGGTCTGACAGTTCG-3′
BIRC5-forward	5′-AGGACCACCGCATCTCTACAT-3′
BIRC5-reverse	5′-AAGTCTGGCTCGTTCTCAGTG-3′
GLS-forward	5′-AGGGTCTGTTACCTAGCTTGG-3′
GLS-reverse	5′-ACGTTCGCAATCCTGTAGATTT-3′
SGK1-forward	5′-AGGATGGGTCTGAACGACTTT-3′
SGK1-reverse	5′-GCCCTTTCCGATCACTTTCAAG-3′
GAPDH-forward	5′-ACTTTGGTATCGTGGAAGGACTCAT-3′
GAPDH-reverse	5′-GTTTTTCTAGACGGCAGGTCAGG-3′

### Western blotting

Antibodies for ERK1/2 (#4695, 1:1000), phospho-ERK1/2 (Thr202/Tyr204) (#4370, 1:1000), LATS1(#3477, 1:1000), LATS2(#5888, 1:1000), p-LATS1/2 (Thr1079) (#8654, 1:1000) and YAP1 (#8418, 1:1000) were purchased from cell signaling technology (Beverly, MA). GAPDH mouse monoclonal antibody (KC-5G4, 1:10000) was obtained from Kangchen (Shanghai, China). Secondary HRP-conjugated antibodies against mouse (SA-00001-1, 1:10000) and rabbit (SA-00001-2, 1:10000) were products of ProteinTech (Chicago, IL). Protein lysates were prepared with RIPA lysis buffer (Beyotime, Shanghai, China). The concentration of protein lysates was determined with a BCA Protein Assay Kit (Pierce; Thermo Fisher Scientific, Rockford, IL). For western blotting, lysates with 20 μg protein per lane were loaded on an 8% SDS-PAGE gel. The proteins were separated and transferred to a PVDF membrane. The membrane was then blocked in 5% non-fat milk at room temperature for 1 h. After that, the membrane was incubated with primary antibodies overnight at 4°C On the next day, the membrane was incubated with indicated secondary antibody at room temperature for 1 h. The blots were developed with SuperSignal West Femto Maximum Sensitivity Substrate (Pierce; Thermo Fisher Scientific).

### Immunofluorescence

T47D cells were grown on glass slide in a 24-well plate. After transfection of control siRNA or ERK1 siRNA and incubation for 72 h, the immunofluorescence was performed as we published before [37]. The nuclei were stained with DAPI, while YAP antibody (#14074, 1: 200, Cell Signaling Technology) was used to detect YAP location.

### Lentivirus mediated knockdown of ERK1

For stable knockdown of ERK1, control scramble shRNA and ERK1 shRNA (1-3 sequences) were cloned into pLKO.1 plasmid. The sequences were: control shRNA: 5′-CCGGTAAGGCTATGAAGAGATACCTCGAGGTATCTCTTCATAGCCTTATTTTTG-3′, ERK1 shRNA1: 5′-CCGGTCATCGGCATCCGAGACATTCCTCGAGGAATGTCTCGGATGCCGATGATTTTTG-3′, ERK1 shRNA2: 5′-CCGGGACAGACATCTCTGCACCCTGCTCGAGCAGGGTGCAGAGATGTCTGTCTTTTTG-3′, ERK1 shRNA3: 5′-CCGGCAACATGAAGGCCCGAAACTACTCGAGTAGTTTCGGGCCTTCATGTTGTTTTTG-3′. 293T cells were transfected with the lentivirus vector and packaging plasmids. At 48 h after transfection, viruses were collected and replaced with fresh medium. At 12 h after incubation, the viruses were collected again and used for infection of T47D cells. Stably infected T47D cells were selected with 2 μg/mL puromycin (InvivoGen, San Diego, CA) for 3 days.

### TEAD activity assay

The activity of TEAD transcription factor was detected with the luciferase reporter assay. The 8×GTIIC-luciferase plasmid (Addgene, Cambridge, MA) and Renilla luciferase pRL-TK plasmid (Promega, Madison, WI) were co-transfected into T47D and MCF7 cells with Lipofectamine 3000 (Invitrogen) following manufacturer’s protocol. After 48 h, cells were harvested, the firefly and renilla luciferase activities were measured by using the Dual-Luciferase Reporter Assay Kit (Promega) according to the manufacturer's instructions. The firefly luciferase was normalized to Renilla luciferase, and the relative firefly activity of ERK1 siRNA group was compared to control group to manifest the relative transcription activity of TEAD.

### Cell viability assay

The cell viability was determined using a CCK-8 kit (Dojindo, Shiga, Japan) following manufacturer’s protocol. Briefly, 10 μL CCK-8 solution was added into indicated well and sustained for 2 h. After that, the medium was transferred into wells of a new 96-well plate. The absorbance at 450 nM was detected to manifest the cell viability.

### CHX chase assay

The cycloheximide (CHX) chase assay was used to analyze the stability of protein. CHX was purchased from MedChemExpress (Monmouth Junction, NJ). Cells were firstly transfected with ERK1 siRNA, ERK2 siRNA or control siRNA for 72 h, CHX (50 μg/ml) was then added into medium for indicated hours. The YAP1 protein levels were detected by Western blotting.

### Gene set enrichment analysis and correlation analyses

The gene expression profile of 50 breast cancer cell lines were retrieved from a previously published work [38]. Cell lines were divided into 2 groups, ie, ERK1 high expression group and ERK1 low expression group. For breast tumors, TCGA (PanCancer Atlas) containing 1082 tumor samples were downloaded and categorized by expression above (high) or below (low) median ERK1 expression by quartiles. Gene set enrichment analysis was performed using GSEA software. A total of 189 gene sets of the oncogenic signature C6 from the Molecular Signatures database (http://www.broadinstitute.org/gsea/msigdb/genesets.jsp?collection=C6, Broad Institute, MSigDB, Version 4.0) were used for the analysis with default settings and 1,000 gene set permutations. One gene set was filtered out by its size (<15 or >500 genes) and excluded from the analysis. Correlation between gene mRNA expression in the gene set of cell lines or human breast tumors were analyzed by calculating the Pearson correlation coefficient. Heat maps were generated using Morpheus software (https://software.broadinstitute.org/morpheus).

### Bioinformatic analysis

For analysis of ERK1, ERK2 and YAP1 expression in breast cancer cell lines, we retrieved mRNA expression data of 50 breast cancer cell lines from a previously published work [38]. In addition, western blotting data of ERK1 and ERK2 in 32 breast cancer cell lines were downloaded and analyzed with Image J software.

To evaluate the expression pattern of ERK1 and ERK2, the human breast tumor microarray data set was obtained from the NCBI-GEO database (GSE18229-GPL1390) containing 16 normal breast samples and 180 tumor samples with subtype classification according to Prat et al [39]. For further confirmation, the TCGA (Nature, 2012) dataset including expression data of 519 breast tumor samples with PAM50 subtype classification were downloaded using cBioPortal [40].

To assess the prognostic value of ERK1 and ERK2, Kaplan-Meier Plotter (KM plotter) database [41] (http://kmplot.com/analysis/index.php?p=service&cancer=breast) was used to explore the association between ERK1/2 expression and breast cancer patient outcome. Cases were divided by expression which was above (high) or below (low) median ERK1 (212046_x_at) or ERK2 (212271_at) expression.

### *In vivo* xenograft experiments

MCF-7 cells (1X10^7^) were transfected with indicated shRNAs (control shRNA, ERK1 shRNA1, ERK1 shRNA1 + YAP1 shRNA), counted, suspended in PBS, and mixed with Matrigel (1:1, BD Biosciences). Female athymic STOCK-Foxn1nu/Nju mice were purchased from Model Animal Research Center of Nanjing University. Mice were randomly separated into 3 groups (n=4) and the PBS–Matrigel mixture were injected into the mammary fat pads. A 0.72-mg E2 60-day release pellet (Innovative Research of America, Sarasota, FL) was implanted subcutaneously on the dorsal side of each mouse a day before injection of tumor cells. Tumor volume was measured every week from the time when tumor was palpable (2 weeks later) until the mice were sacrificed (week 6). Tumor volumes were calculated by measuring width and length of tumors with a Vernier caliper and calculated by formula as listed: Volume = (Length × Width × Width)/2. All animal studies were performed in accordance with the guidelines for the care and use of laboratory animals of Nanjing Normal University.

### Statistical analysis

All data were analyzed with Graphpad Prism 6.0 and presented as mean ± SD. The differences between two groups were analyzed using Student’s t test. The differences among three groups were analyzed using one-way ANOVA followed by Newman Keuls analysis. A p value less than 0.05 was considered statistically significant. All experiments were repeated at least 3 times.

## Supplementary Material

Supplementary Figures

Supplementary Table 1

## References

[1] Bray F, Ferlay J, Soerjomataram I, Siegel RL, Torre LA, Jemal A. Global cancer statistics 2018: GLOBOCAN estimates of incidence and mortality worldwide for 36 cancers in 185 countries. CA Cancer J Clin. 2018; 68:394–424. 10.3322/caac.21492 30207593

[2] Tsimberidou AM. Targeted therapy in cancer. Cancer Chemother Pharmacol. 2015; 76:1113–32. 10.1007/s00280-015-2861-1 26391154 PMC4998041

[3] Sun Y, Liu WZ, Liu T, Feng X, Yang N, Zhou HF. Signaling pathway of MAPK/ERK in cell proliferation, differentiation, migration, senescence and apoptosis. J Recept Signal Transduct Res. 2015; 35:600–04. 10.3109/10799893.2015.1030412 26096166

[4] Johnson GL, Lapadat R. Mitogen-activated protein kinase pathways mediated by ERK, JNK, and p38 protein kinases. Science. 2002; 298:1911–12. 10.1126/science.1072682 12471242

[5] Sebolt-Leopold JS, Herrera R. Targeting the mitogen-activated protein kinase cascade to treat cancer. Nat Rev Cancer. 2004; 4:937–47. 10.1038/nrc1503 15573115

[6] Peng WX, Huang JG, Yang L, Gong AH, Mo YY. Linc-RoR promotes MAPK/ERK signaling and confers estrogen-independent growth of breast cancer. Mol Cancer. 2017; 16:161. 10.1186/s12943-017-0727-3 29041978 PMC5645922

[7] Burotto M, Chiou VL, Lee JM, Kohn EC. The MAPK pathway across different malignancies: a new perspective. Cancer. 2014; 120:3446–56. 10.1002/cncr.28864 24948110 PMC4221543

[8] Ma L, Li K, Guo Y, Sun X, Deng H, Li K, Feng Q, Li S. Ras-Raf-MAPK signaling promotes nuclear localization of FOXA transcription factor SGF1 via Ser91 phosphorylation. Biochim Biophys Acta Mol Cell Res. 2018; 1865:560–71. 10.1016/j.bbamcr.2018.01.007 29355586

[9] Lloyd AC. Distinct functions for ERKs? J Biol. 2006; 5:13. 10.1186/jbiol46 16879721 PMC1781519

[10] Frémin C, Saba-El-Leil MK, Lévesque K, Ang SL, Meloche S. Functional Redundancy of ERK1 and ERK2 MAP Kinases during Development. Cell Rep. 2015; 12:913–21. 10.1016/j.celrep.2015.07.011 26235619

[11] Vantaggiato C, Formentini I, Bondanza A, Bonini C, Naldini L, Brambilla R. ERK1 and ERK2 mitogen-activated protein kinases affect Ras-dependent cell signaling differentially. J Biol. 2006; 5:14. 10.1186/jbiol38 16805921 PMC1781522

[12] Harvey KF, Zhang X, Thomas DM. The Hippo pathway and human cancer. Nat Rev Cancer. 2013; 13:246–57. 10.1038/nrc3458 23467301

[13] Meng Z, Moroishi T, Guan KL. Mechanisms of Hippo pathway regulation. Genes Dev. 2016; 30:1–17. 10.1101/gad.274027.115 26728553 PMC4701972

[14] Park JH, Shin JE, Park HW. The Role of Hippo Pathway in Cancer Stem Cell Biology. Mol Cells. 2018; 41:83–92. 29429151 10.14348/molcells.2018.2242 PMC5824027

[15] Zhao B, Li L, Tumaneng K, Wang CY, Guan KL. A coordinated phosphorylation by Lats and CK1 regulates YAP stability through SCF(beta-TRCP). Genes Dev. 2010; 24:72–85. 10.1101/gad.1843810 20048001 PMC2802193

[16] Mo JS, Park HW, Guan KL. The Hippo signaling pathway in stem cell biology and cancer. EMBO Rep. 2014; 15:642–56. 10.15252/embr.201438638 24825474 PMC4197875

[17] Shi J, Li F, Yao X, Mou T, Xu Z, Han Z, Chen S, Li W, Yu J, Qi X, Liu H, Li G. The HER4-YAP1 axis promotes trastuzumab resistance in HER2-positive gastric cancer by inducing epithelial and mesenchymal transition. Oncogene. 2018; 37:3022–38. 10.1038/s41388-018-0204-5 29535422 PMC5978807

[18] Wang Y, Justilien V, Brennan KI, Jamieson L, Murray NR, Fields AP. PKCι regulates nuclear YAP1 localization and ovarian cancer tumorigenesis. Oncogene. 2017; 36:534–45. 10.1038/onc.2016.224 27321186 PMC5173453

[19] Sun D, Li X, He Y, Li W, Wang Y, Wang H, Jiang S, Xin Y. YAP1 enhances cell proliferation, migration, and invasion of gastric cancer in vitro and in vivo. Oncotarget. 2016; 7:81062–76. 10.18632/oncotarget.13188 27835600 PMC5348376

[20] Wen Y, Ji Y, Zhang Y, Jiang B, Tang C, Wang Q, Chen X, Jia L, Gu W, Xu X. Knockdown of Yes-Associated Protein Induces the Apoptosis While Inhibits the Proliferation of Human Periodontal Ligament Stem Cells through Crosstalk between Erk and Bcl-2 Signaling Pathways. Int J Med Sci. 2017; 14:1231–40. 10.7150/ijms.20504 29104479 PMC5666556

[21] Cordenonsi M, Zanconato F, Azzolin L, Forcato M, Rosato A, Frasson C, Inui M, Montagner M, Parenti AR, Poletti A, Daidone MG, Dupont S, Basso G, et al. The Hippo transducer TAZ confers cancer stem cell-related traits on breast cancer cells. Cell. 2011; 147:759–72. 10.1016/j.cell.2011.09.048 22078877

[22] Buscà R, Pouysségur J, Lenormand P. ERK1 and ERK2 Map Kinases: Specific Roles or Functional Redundancy? Front Cell Dev Biol. 2016; 4:53. 10.3389/fcell.2016.00053 27376062 PMC4897767

[23] Lei Q, Tan J, Yi S, Wu N, Wang Y, Wu H. Mitochonic acid 5 activates the MAPK-ERK-yap signaling pathways to protect mouse microglial BV-2 cells against TNFα-induced apoptosis via increased Bnip3-related mitophagy. Cell Mol Biol Lett. 2018; 23:14. 10.1186/s11658-018-0081-5 29636771 PMC5887257

[24] Lu C, Chen X, Wang Q, Xu X, Xu B. TNFα promotes glioblastoma A172 cell mitochondrial apoptosis via augmenting mitochondrial fission and repression of MAPK-ERK-YAP signaling pathways. Onco Targets Ther. 2018; 11:7213–27. 10.2147/OTT.S184337 30425514 PMC6203110

[25] Hülter-Hassler D, Wein M, Schulz SD, Proksch S, Steinberg T, Jung BA, Tomakidi P. Biomechanical strain-induced modulation of proliferation coincides with an ERK1/2-independent nuclear YAP localization. Exp Cell Res. 2017; 361:93–100. 10.1016/j.yexcr.2017.10.006 29017756

[26] You B, Yang YL, Xu Z, Dai Y, Liu S, Mao JH, Tetsu O, Li H, Jablons DM, You L. Inhibition of ERK1/2 down-regulates the Hippo/YAP signaling pathway in human NSCLC cells. Oncotarget. 2015; 6:4357–68. 10.18632/oncotarget.2974 25738359 PMC4414195

[27] Krens SF, Corredor-Adámez M, He S, Snaar-Jagalska BE, Spaink HP. ERK1 and ERK2 MAPK are key regulators of distinct gene sets in zebrafish embryogenesis. BMC Genomics. 2008; 9:196. 10.1186/1471-2164-9-196 18442396 PMC2390552

[28] Lehmann W, Mossmann D, Kleemann J, Mock K, Meisinger C, Brummer T, Herr R, Brabletz S, Stemmler MP, Brabletz T. ZEB1 turns into a transcriptional activator by interacting with YAP1 in aggressive cancer types. Nat Commun. 2016; 7:10498. 10.1038/ncomms10498 26876920 PMC4756710

[29] Kim T, Yang SJ, Hwang D, Song J, Kim M, Kyum Kim S, Kang K, Ahn J, Lee D, Kim MY, Kim S, Seung Koo J, Seok Koh S, et al. A basal-like breast cancer-specific role for SRF-IL6 in YAP-induced cancer stemness. Nat Commun. 2015; 6:10186. 10.1038/ncomms10186 26671411 PMC4703869

[30] Kang W, Tong JH, Lung RW, Dong Y, Zhao J, Liang Q, Zhang L, Pan Y, Yang W, Pang JC, Cheng AS, Yu J, To KF. Targeting of YAP1 by microRNA-15a and microRNA-16-1 exerts tumor suppressor function in gastric adenocarcinoma. Mol Cancer. 2015; 14:52. 10.1186/s12943-015-0323-3 25743273 PMC4342823

[31] Liu G, Huang K, Jie Z, Wu Y, Chen J, Chen Z, Fang X, Shen S. CircFAT1 sponges miR-375 to promote the expression of Yes-associated protein 1 in osteosarcoma cells. Mol Cancer. 2018; 17:170. 10.1186/s12943-018-0917-7 30514309 PMC6280518

[32] Kim TD, Jin F, Shin S, Oh S, Lightfoot SA, Grande JP, Johnson AJ, van Deursen JM, Wren JD, Janknecht R. Histone demethylase JMJD2A drives prostate tumorigenesis through transcription factor ETV1. J Clin Invest. 2016; 126:706–20. 10.1172/JCI78132 26731476 PMC4731184

[33] Melhuish TA, Kowalczyk I, Manukyan A, Zhang Y, Shah A, Abounader R, Wotton D. Myt1 and Myt1l transcription factors limit proliferation in GBM cells by repressing YAP1 expression. Biochim Biophys Acta Gene Regul Mech. 2018; 1861:983–95. 10.1016/j.bbagrm.2018.10.005 30312684 PMC6203443

[34] Zanconato F, Cordenonsi M, Piccolo S. YAP/TAZ at the Roots of Cancer. Cancer Cell. 2016; 29:783–803. 10.1016/j.ccell.2016.05.005 27300434 PMC6186419

[35] Mirzoeva OK, Das D, Heiser LM, Bhattacharya S, Siwak D, Gendelman R, Bayani N, Wang NJ, Neve RM, Guan Y, Hu Z, Knight Z, Feiler HS, et al. Basal subtype and MAPK/ERK kinase (MEK)-phosphoinositide 3-kinase feedback signaling determine susceptibility of breast cancer cells to MEK inhibition. Cancer Res. 2009; 69:565–72. 10.1158/0008-5472.CAN-08-3389 19147570 PMC2737189

[36] Dennis G Jr, Sherman BT, Hosack DA, Yang J, Gao W, Lane HC, Lempicki RA. DAVID: Database for Annotation, Visualization, and Integrated Discovery. Genome Biol. 2003; 4:3. 10.1186/gb-2003-4-5-p312734009

[37] Yu S, Cai X, Wu C, Wu L, Wang Y, Liu Y, Yu Z, Qin S, Ma F, Thiery JP, Chen L. Adhesion glycoprotein CD44 functions as an upstream regulator of a network connecting ERK, AKT and Hippo-YAP pathways in cancer progression. Oncotarget. 2015; 6:2951–65. 10.18632/oncotarget.3095 25605020 PMC4413630

[38] Neve RM, Chin K, Fridlyand J, Yeh J, Baehner FL, Fevr T, Clark L, Bayani N, Coppe JP, Tong F, Speed T, Spellman PT, DeVries S, et al. A collection of breast cancer cell lines for the study of functionally distinct cancer subtypes. Cancer Cell. 2006; 10:515–27. 10.1016/j.ccr.2006.10.008 17157791 PMC2730521

[39] Prat A, Parker JS, Karginova O, Fan C, Livasy C, Herschkowitz JI, He X, Perou CM. Phenotypic and molecular characterization of the claudin-low intrinsic subtype of breast cancer. Breast Cancer Res. 2010; 12:R68. 10.1186/bcr2635 20813035 PMC3096954

[40] Cerami E, Gao J, Dogrusoz U, Gross BE, Sumer SO, Aksoy BA, Jacobsen A, Byrne CJ, Heuer ML, Larsson E, Antipin Y, Reva B, Goldberg AP, et al. The cBio cancer genomics portal: an open platform for exploring multidimensional cancer genomics data. Cancer Discov. 2012; 2:401–04. 10.1158/2159-8290.CD-12-0095 22588877 PMC3956037

[41] Lánczky A, Nagy Á, Bottai G, Munkácsy G, Szabó A, Santarpia L, Győrffy B. miRpower: a web-tool to validate survival-associated miRNAs utilizing expression data from 2178 breast cancer patients. Breast Cancer Res Treat. 2016; 160:439–46. 10.1007/s10549-016-4013-7 27744485

