# Supplementary material for: ERK1 indicates good prognosis and inhibits breast cancer progression by suppressing YAP1 signaling
Source: Aging (Albany NY). 2019 Dec 18;11(24):12295–314. doi: 10.18632/aging.102572 (PMC6949071; doi:10.18632/aging.102572)
Supplement: Supplementary Figures [file aging-11-102572-s002..pdf]

SUPPLEMENTARY FIGURES

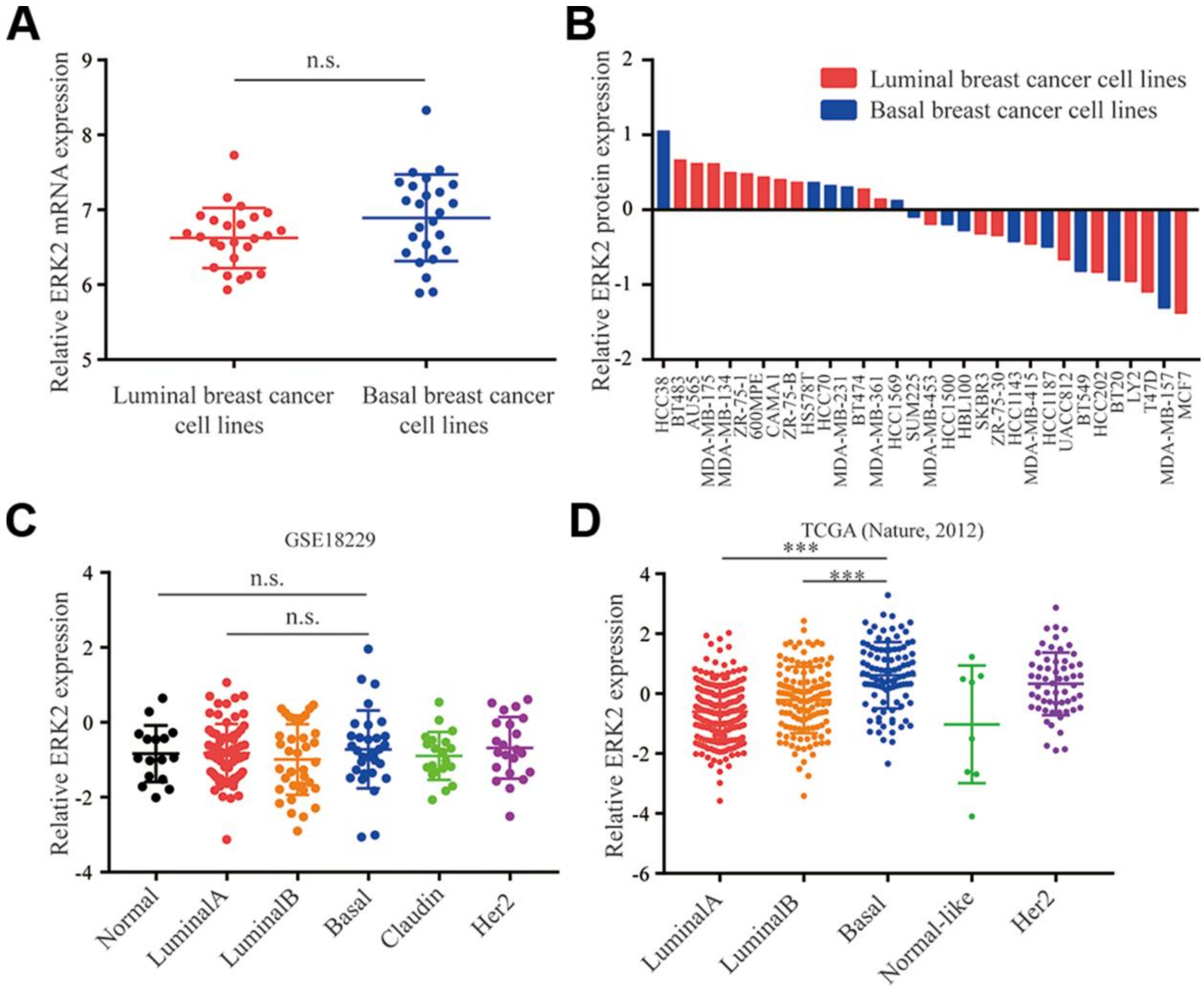

Supplementary Figure 1. Expression pattern of ERK2 in breast cancer. (**A**) Analysis of data for 50 breast cancer cell lines suggested that ERK2 mRNA levels were similar in luminal breast cancer and basal subtype. (**B**) Analysis of western blotting data from 32 breast cancer cell lines suggested that ERK2 protein expression was similar in luminal breast cancer when compared with basal subtype. (**C**) Analysis of GSE18229 dataset for 16 normal breast and 180 breast tumors suggested that ERK2 levels were similar in basal breast tumors when compared with LuminalA subtype and normal breast tissues. (**D**) Analysis of TCGA dataset for 519 breast tumors showed that ERK2 was upregulated in basal breast tumors when compared with LuminalA and LuminalB subtypes. ***, $p<0.001$.

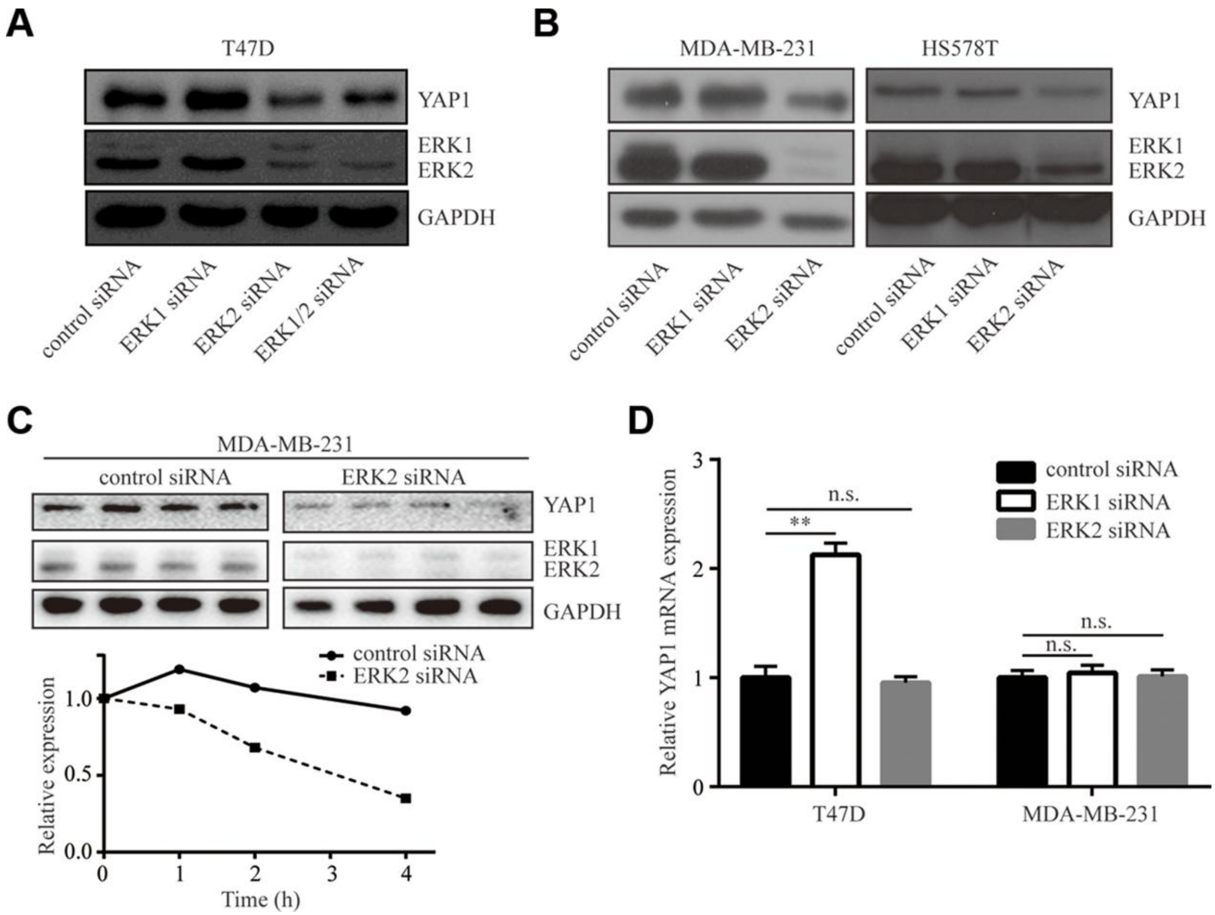

Supplementary Figure 2. ERK2 regulated YAP1 protein stability in breast cancer cells. (**A**) While ERK1 silencing increased YAP1 protein expression, silencing of ERK2 decreased YAP1 protein levels in T47D cells. (**B**) Silencing of ERK2 decreased YAP1 protein levels in MDA-MB-231 and HS578T cells. (**C**) The CHX chase assay indicated that silencing of ERK2 destabilized YAP1 in MDA-MB-231 cells. (**D**) Silencing of ERK2 did not changed YAP1 mRNA expression in T47D and MDA-MB-231 cells. **, p<0.01.
